# A case of charcot's feet in a patient with parkinson's disease: a case report

**DOI:** 10.1186/1757-1626-2-187

**Published:** 2009-11-09

**Authors:** Amresh P Singh, Andrew J Kelly

**Affiliations:** 1 Trauma and Orthopaedics, Musgrove Park Hospital, Taunton, TA2 5DA, UK

## Abstract

**Introduction:**

Parkinson's Disease (PD), amongst its motor symptoms, can cause dystonia of the limbs and trunk. This can lead to subsequent deformities.

**Case Presentation:**

We describe a case where Parkinson's Disease has gone on to cause bilateral Charcot feet with rocker-bottom deformity.

**Conclusion:**

There is recognised pattern of foot deformities seen in Parkinson's disease and it is important to realise that patients may go on to develop a Charcot joint. This would warrant early referral to an orthopaedic surgeon so that appropriate management can limit joint damage and deformity.

## Background

Charcot's arthropathy is a progressive condition characterized by joint destruction, debilitating deformities and pathological fractures. Though it can occur at any joint, it is most commonly seen in the lower extremity, at the foot and ankle. Historically the most common cause was syphilis but diabetes is now the most common aetiology. The major theories to explain Charcot joints are the Neurotraumatic and Neurovascualar theory. The first suggests that the arthropathy is caused by unperceived trauma in an insensate foot and the latter, that there is an autonomic neuropathy causing a mismatch in bone destruction and synthesis. Amongst its other complications, Charcot arthropathy can lead to midfoot collapse and inversion of the plantar arch leading to a rocker-bottom foot deformity [1].

## Case presentation

**Figure 1:**
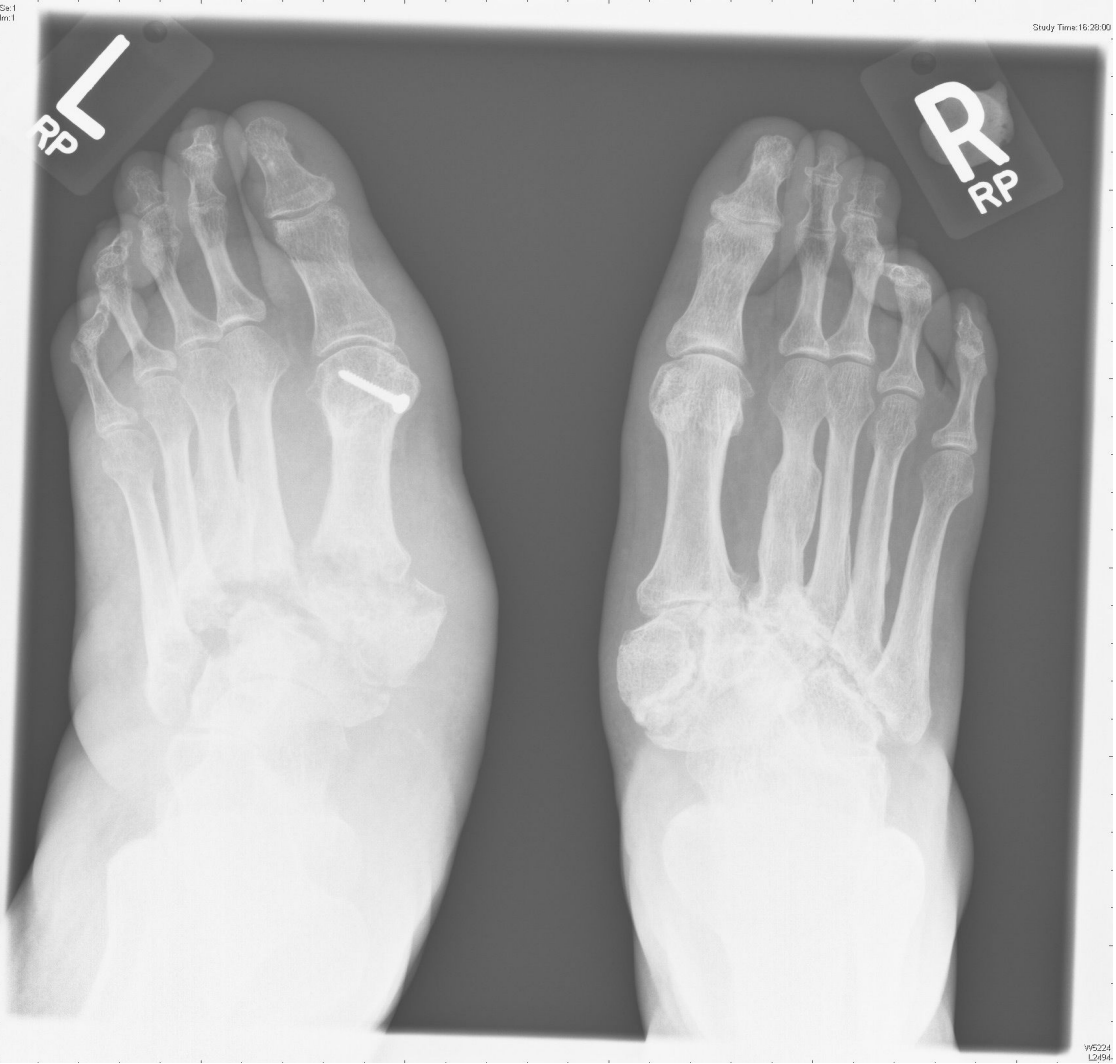
**Radiograph demonstrating bilateral midfoot collapse**.

We present a case of a 58-year-old female patient (Caucasian British) with Parkinson's disease. This was initially diagnosed in 1994 by a neurologist based on her clinical presentation. In 2004, she presented to the Orthopaedic surgeons with a 6 month history of right sided foot pain and loss of foot shape. The foot had been swollen but this had started to settle. Examination revealed a warm and swollen foot, abducted forefoot and a flat longitudinal arch. Radiographs taken at this time showed disruption of the tarsometatarsal joint with dorsolateral subluxation and bone fragmentation. There was also evidence of an old 2^nd ^and 4^th ^metatarsal fracture. A diagnosis of Tarsometatarsal Charcot Arthropathy was made. She was treated with an aircast boot to prevent further deterioration of alignment. In early 2006, the left foot also showed the early stages of Charcot Arthropathy and by the end of the year she had developed bilateral midfoot collapse. (Figure 1) An electromyogram of the lower limbs did not show any evidence of peripheral neuropathy or L5/S1 radiculopathy and this was also confirmed with a neurology opinion. By 2008, she had unfortunately developed bilateral rocker-bottom feet despite treatment with total contact casts. She was unable to tolerate this and is currently being treated in an aircast walker.

## Discussion

James Parkinson first described the symptoms of the "shaking palsy" in 1812. Unfortunately, Parkinson made no mention of the rigidity or the mask like facies. It was Jean-Martin Charcot, who felt that Parkinson was the crucial pioneer and named the condition "Maladie de Parkinson." He favoured this over the original name "Paralysis Agitans" [2,3].

This case of is of particular interest as, in the literature search preformed by the authors, there are no reported cases of Charcot feet in a patient with PD and for the historical link mentioned above. There are, however, other foot abnormalities associated with PD. These are seen in 12.8% of patients with PD. (ashour 2006) Charcot first described abnormal postures of the feet in 1877 [4]. He noted the following deformities:

1. Talipes equinovarus

2. Hyperextension of the hallux

3. Clawing of the lesser toes

These deformities where placed under the phrase "striatal foot" by Charcot. The term "striatal" being used as it referred to pathology in the neostriatum which was suggested to be the cause. Early reports attributed the postural abnormalities to muscular rigidity but subsequent studies suggest an extrapyramidal origin. Dystonias associated with PD can affect the hand, feet, trunk (camptocormia), head (anterocollis) and cause scoliosis. There are many theories as to the cause of these dystonias, such as damage to the striatopallidothalamic pathway, caudate nucleus, globus pallidus and putamen. The exact mechanism, however, is unclear [5].

Our patient has no evidence of diabetes mellitus and had an EMG to exclude a peripheral neuropathy. She also did not have the typical deformities seen in PD. We postulate that the dystonia, as a result of her PD, would have resulted in altered feet posture and gait. The subsequent altered mechanics may have caused abnormal stresses in the joints and the resulting trauma initiated the Charcot arthropathy.

## Conclusion

Though there is recognised pattern of foot deformities seen in Parkinson's disease, it is important to realise that such patients may go on to develop a Charcot joint. This would warrant early referral to an orthopaedic surgeon so that appropriate management can be put in place to prevent or limit joint damage and deformity. We would aim to limit functional disability in patients already coping with PD symptoms.

## Consent

Written informed consent was obtained from the patient for publication of this case report and accompanying images. A copy of the written consent is available for review by the Editor-in-Chief of this journal.

## Competing interests

The authors declare that they have no competing interests.

## Authors' contributions

"APS analyzed the patient's notes and images regarding the details of their Parkinson's disease and the development of their subsequent feet deformities. APS carried out a literature search on these topics. AJK is the consultant responsible for this patient and supervised the writing of this report. All authors read and approved the final manuscript."
